# Unsupervised Event Graph Representation and Similarity Learning on Biomedical Literature

**DOI:** 10.3390/s22010003

**Published:** 2021-12-21

**Authors:** Giacomo Frisoni, Gianluca Moro, Giulio Carlassare, Antonella Carbonaro

**Affiliations:** 1 Department of Computer Science and Engineering (DISI), University of Bologna, 40126 Bologna, Italy; giacomo.frisoni@unibo.it (G.F.); gianluca.moro@unibo.it (G.M.); 2 Independent Researcher, 48018 Faenza, Italy; giulio.carlassare@gmail.com

**Keywords:** event embedding, graph representation learning, graph similarity learning, metric learning, graph kernels, graph neural networks, event extraction, biomedical text mining

## Abstract

The automatic extraction of biomedical events from the scientific literature has drawn keen interest in the last several years, recognizing complex and semantically rich graphical interactions otherwise buried in texts. However, very few works revolve around learning embeddings or similarity metrics for event graphs. This gap leaves biological relations unlinked and prevents the application of machine learning techniques to promote discoveries. Taking advantage of recent deep graph kernel solutions and pre-trained language models, we propose Deep Divergence Event Graph Kernels (DDEGK), an unsupervised inductive method to map events into low-dimensional vectors, preserving their structural and semantic similarities. Unlike most other systems, DDEGK operates at a graph level and does not require task-specific labels, feature engineering, or known correspondences between nodes. To this end, our solution compares events against a small set of anchor ones, trains cross-graph attention networks for drawing pairwise alignments (bolstering interpretability), and employs transformer-based models to encode continuous attributes. Extensive experiments have been done on nine biomedical datasets. We show that our learned event representations can be effectively employed in tasks such as graph classification, clustering, and visualization, also facilitating downstream semantic textual similarity. Empirical results demonstrate that DDEGK significantly outperforms other state-of-the-art methods.

## 1. Introduction

Advances in both research and computational methods act as the catalyst for a large number of scientific publications, especially in the biomedical domain [1]. Identifying and tracking the discoveries and theories disseminated within life science papers is critical to speed up medical progress. However, keeping up with all the latest articles has become increasingly challenging for human domain experts. According to bibliometric results verifiable at https://pubmed.ncbi.nlm.nih.gov/ using the query “1700:2021[dp]”—accessed on 11 December 2021—the annual rate of biomedical publications registered on PubMed is growing exponentially. As biology literature increases in the form of text documents, the automatic extraction of biomedical entities (e.g., proteins, genes, diseases, drugs) and their semantic relations have been intensively investigated [2]. Recent developments in deep learning (DL) and natural language processing (NLP) have enabled intelligent ways to uncover structured, concise, and unambiguous knowledge mentioned in large unstructured text corpora. In biomedical text mining, classified named entities and pairwise relations are notoriously insufficient for understanding interactions ranging from molecular-level reactions to organism-level outcomes [3]. As a result, the extraction of complex relations (namely, events) from the text—involving nested structures and multiple participants—has become a quintessential task knotted with semantic parsing. According to the BioNLP-ST competitions [4,5,6], events are composed of a trigger (a textual mention which clearly testifies their occurrence, e.g., “interacts”, “regulates”), a type (e.g., “binding”, “regularization”), and a set of arguments with a specific role, which can be entities or events themselves. Following in the footsteps of NLP breakthroughs, events underpin many valuable applications, such as literature-based knowledge discovery [7], biological network construction [8], pathway curation [9], diagnosis prediction [10], and question answering [11].

**Figure 1:**
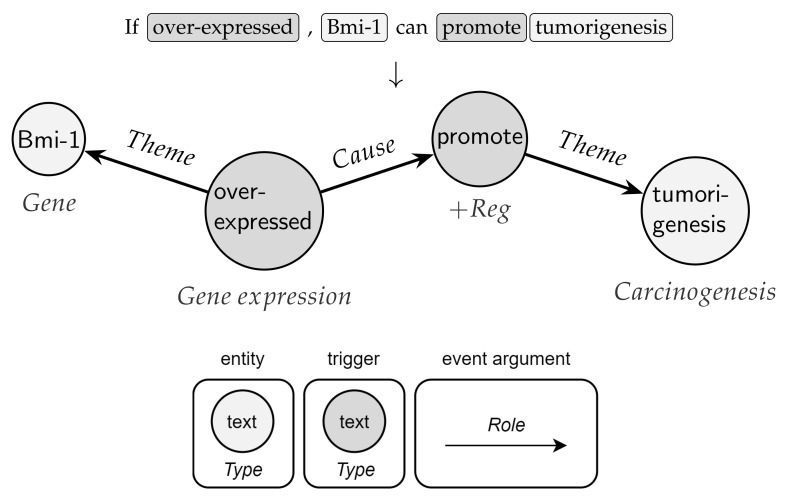
A biomedical event graph extracted from a sentence. The example exhibits two nested events: (i) a Positive regulation event (+Reg), anchored by a trigger “promote”, affecting “tumorigenesis” as *Theme* argument; (ii) a Gene expression of “Bmi-1” with (i) as a *Cause* argument.

Like many other relational data in real-world scenarios, bio-events can be conveniently and naturally modeled as graphs (directed, acyclic, and labeled) [2]. As depicted in Figure 1, nodes are given by triggers (roots) and entities, while edges represent argument roles. In this vein, graphs are a ubiquitous modeling tool with solid mathematical foundations. Notably, the application of deep neural networks on non-Euclidean graph-structured data is an emerging trend [12,13]. Effective graph analytics provide researchers with a deeper understanding of the data, supporting various tasks in multiple domains, including bioinformatics, chemoinformatics, neuroscience, and sociology. These tasks range from protein folding to recommender systems, from social network analysis to molecular studies and drug design [13].

**Figure 2:**
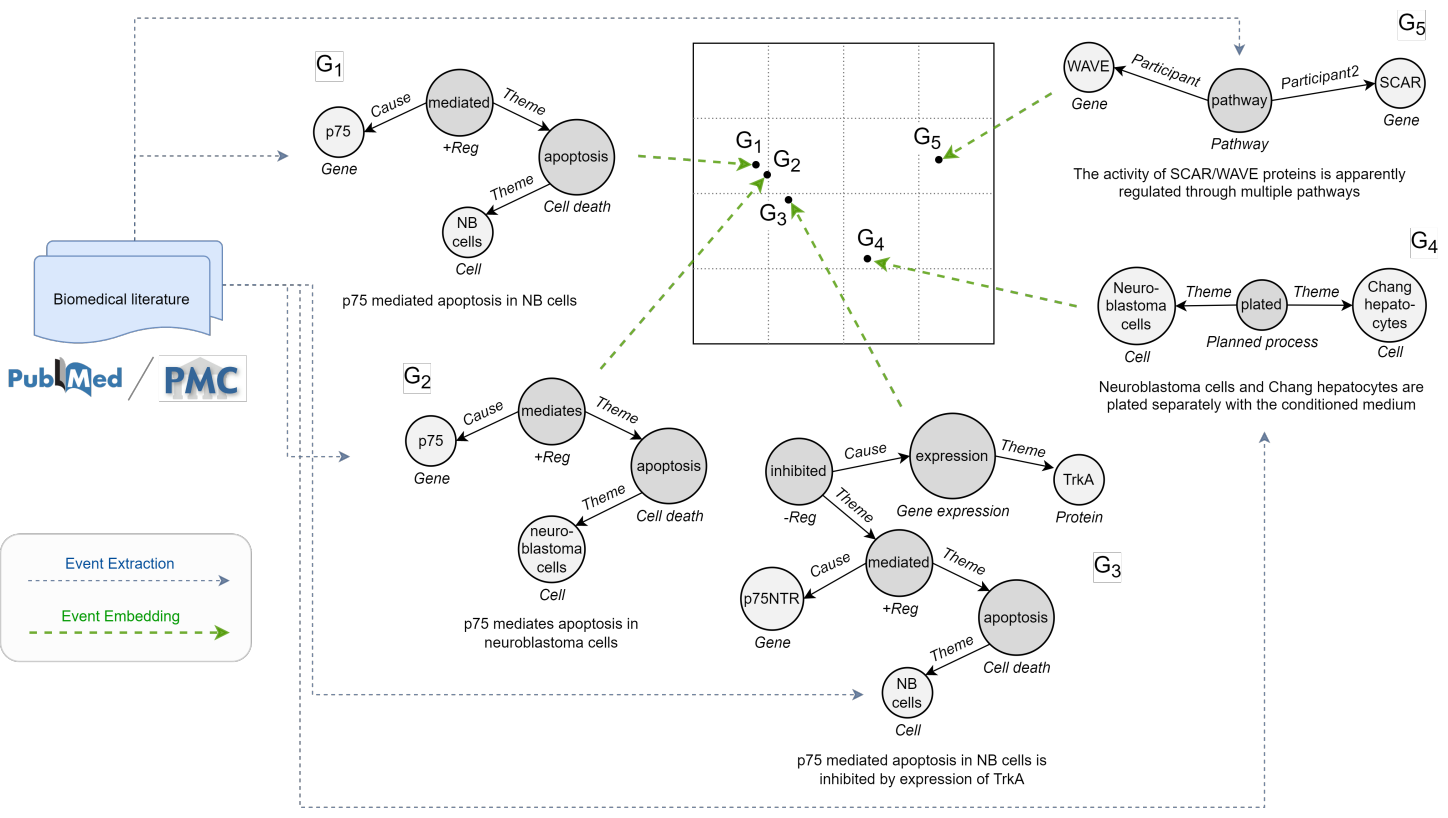
Illustration of similarity-preserving event graph embeddings, with sample events taken from a cancer genetics database (CG13). Each event graph—for which is shown also the textual mention—is mapped into an embedding vector (denoted as a dot in the simplified 2D space). The target embedding function should consider both structure and semantics. The example reports some interesting cases: (i) graphs with same structure and overall semantics ($G_{1}$ and $G_{2}$), where NB is the acronym for neuro-blastoma; (ii) graphs with different structure but similar semantics ($G_{2}$ and $G_{3}$), where the process represented by $G_{2}$ is contained in $G_{3}$; (iii) graphs with same structure but different semantics ($G_{4}$ and $G_{5}$). For precision, we underline that our embeddings are not calculated directly from the raw literature but from events mentioned in abstracts or full texts, mainly obtainable from existing annotations or predictions of event extraction systems (blue arrow).

Leaving aside link predictions and node-, edge-, or graph-level classifications, one of the fundamental problems is to retrieve a set of similar graphs from a database given a user query. The matching, clustering, ranking, and retrieval of biomedical event graphs expressed in the literature can significantly facilitate the work of scientists in exploring the latest developments or identifying relevant studies. For instance, it would allow searching for interactions similar to one provided in input, looking for events involving certain biomedical entities or types of participants with precise roles. Additionally, it would enable the aggregation and quantification of highly related concept units, recognizing the number of times an interaction has been expressed. To solve this task, graph similarity metrics based on predefined distance notions and feature engineering are very costly to compute in practice and hardly generalizable [14]. In the last few years, researchers have therefore formulated graph similarity estimation as a learning problem [15]. Deep graph similarity learning (GSL) uses deep strategies to automatically learn a metric for measuring the similarity scores between graph object pairs. The key idea is training a model that maps input graphs to a target vector space such that the distance in the target space approximates the one between the symbolic representations in the input space (Figure 2). It follows that GSL is strongly interconnected with graph representation learning (GRL). By projecting raw graph data into continuous low-dimensional vector spaces while preserving intrinsic graph properties, the so-called graph embeddings provide a general tool to efficiently store and access relational knowledge in both time and space [16]. These features can promote the resolution of many machine learning problems on graphs via standard frameworks suitable for vectorized representations.

Despite their potential, GSL and GRL contributions dedicated to the event sphere are still scarce and limited. Indeed, the high representational power of event graphs brings with it considerable challenges. First, events are intrinsically discrete objects with an *irregular structure* that can vary significantly across instances, even if associated with the same event type; they are more difficult to analyze than text/image/video/audio defined on regular lattices. Second, there are *no datasets reporting similarity scores between event pairs* for supervised approaches, which can be tedious and time-consuming to produce in large numbers. Nevertheless, existing works mostly fail to utilize unlabeled graph pairs for metric learning. Third, systems should be *interpretable* in the process of predicting similarities; in the biomedical domain, more than others, reliability is a requirement that cannot be ignored. Fourth, as is often the case with labeled graphs [17,18], subtle nuances in labels can make two events semantically agreeing or not, while events with distinct structures can still be similar. Therefore, to be broadly useful, a successful solution should consider *both event graph structure and semantics*, fine-grained (e.g., synonym) and coarse-grained (broad scenario). Fifth, many methods rely on a known correspondence between the nodes of two graphs, becoming impractical in the event realm, where node labels are free text spans and there are multiple ways of referring to the same entity or relation (i.e., *no id sharing*). Sixth, looking closer to GRL, most work focuses on node-, edge-, or community-level representations above a single large input graph. Instead, events extracted from a corpus correspond to many independent and small-sized graphs, primarily asking for *whole-graph embeddings*. Seventh, event representations should be *general*, but present solutions tend to learn intermediate graph and node embeddings only as a precondition to maximize accuracy on a set of narrow classification tasks, giving rise to heavily biased vectors. Finally, we would like not to be bound to a set of graphs known in advance, preferring *inductive* embedding approaches.

This paper addresses the problem of similarity and representation learning for biomedical event graphs. Specifically, we propose an unsupervised method for computing general-purpose event graph representations using deep graph kernels to satisfy all the aforementioned needs. With the term “unsupervised”, we mean models based only on information available in the event graphs (structure and node/edge features), without task-specific labels (e.g., similarity scores) or loss functions. Our work is based on DDGK [19], which is capable of deriving a kernel from structural and semantic divergences between graph pairs without requiring feature engineering, algorithmic insights, similarity labels, structural or domain-specific assumptions (like graph primitives’ importance). Moreover, it includes a cross-graph attention mechanism to probabilistically align node representations, aiding the interpretation of proximities by looking for similar substructures. Guided by event characteristics in the NLP area, we provide an extended version of DDGK, called Deep Divergence Event Graph Kernels (DDEGK), integrating pre-trained language models to capture semantic consistencies among continuous labels.

To sum up, our main contributions are:

- **In-depth literature analysis**. We offer newcomers in the field a global perspective on the problem with insightful discussions and an extensive reference list, also providing systematic taxonomies.
- **Deep Divergence Event Graph Kernels**. A novel method of event graph similarity learning centered on constructing general event embeddings with deep graph kernels, considering both structure and semantics.
- **Experimental results**. We conduct extensive experiments to demonstrate the effectiveness of DDEGK in real-world scenarios. We show that our solution successfully recognizes fine- and coarse-grained similarities between biomedical events. Precisely, when used as features, the event representations learned by DDEGK achieve new state-of-the-art or competitive results on different extrinsic evaluation tasks, comprising sentence similarity, event classification, and clustering. To shed light on the performance of different embedding techniques, we compare with a rich set of baselines on nine datasets having distinct biological views.

The rest of the paper is organized as follows: First, in Section 2, we examine related work, outlining a bird’s-eye view of the research context. Then, to provide a solid foundation, Section 3 introduces notation and preliminary concepts necessary to understand our study. Next, Section 4 details the datasets and clearly describes the architecture and operation of DDEGK, while Section 5 presents the experiments and the results obtained. Section 6 explains findings, limits, and applicability. Finally, Section 7 closes the discussion and points out future directions.

## 2. Related Work

**Figure 3:**
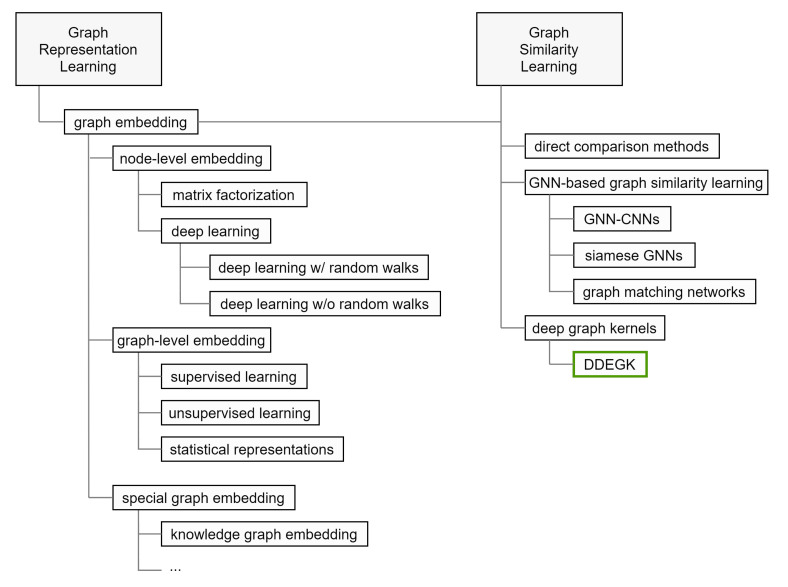
Proposed taxonomy for categorizing the literature on graph representation and similarity learning. The green box highlights the position of DDEGK.

This section accurately positions our work, outlines its scope, surveys related topics, and compares it with various methods. Our main objective is to give the reader an overview of the research done so far, highlighting the open issues that motivate the presented article. In Figure 3, we characterize the research space that orbits around DDEGK.

### 2.1. Individual Graph Embedding

Dealing with relational data requires significant computational resources, domain expertise, and task-dependent features to incorporate structural information into predictive models. Graph embedding effectively solves the high computation and space costs of graph analytics, combining the benefits of statistical and structural approaches.

**Figure 4:**
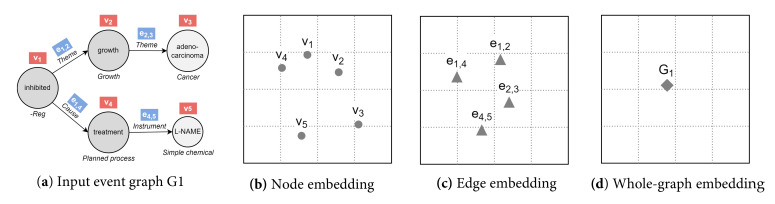
Example of embedding an event graph (**a**) into a 2D space with different output granularities: node-level (**b**), edge-level (**c**), and graph-level (**d**). We achieve (**d**) based on inter-graph node similarities. Knowledge graph embedding typically mixes nodes and edges in the same space, i.e., a combination of (**b**,**c**). For visual clarity, node and edge identifiers are shown within red and blue boxes, respectively.

Nowadays, a plethora of graph embedding procedures has been released in different streams of literature. It provides powerful tools to construct vectorized feature spaces for graphs and their components. Graph similarity methods based on graph embedding seek to utilize learned representations for predicting similarity scores. Below, we summarize the approaches where embeddings are learned independently between graphs, in a separate stage prior to similarity estimation. We divide our analysis in consonance with the embedding output (embedding granularity, see Figure 4), the methodological approach, and the nature of the input graphs.

Since most models refer to specific graph use-cases, we clarify the problem settings as a premise. In this paper, we use the term “event” to refer to complex interactions resulting from the closed-domain event extraction task, where target types are predefined. Pointedly, events are heterogeneous and unweighted graphs with a lot of auxiliary information. Nodes and edges (i.e., triggers/entities and argument roles) belong to different types, defined in an ontological schema and serving as categorical labels. Furthermore, each node has a textual attribute with a continuous value expressing the text span that allowed its recognition. Intuitively, node/edge class labels and node text features provide more details about the event graph, potentially leading to better embeddings: they correlate for close instances and support inductive learning (i.e., generalization towards unseen samples or domains).

#### 2.1.1. Node Embedding

Most existing research on GRL focuses on node-level embedding.

Matrix Factorization. Historically, the pioneering (and best-studied theoretically) models in graph embeddings are factorization-based. They represent graph properties—such as adjacencies or node pairwise similarities—in the form of a large matrix and try to approximate it with a low-rank matrix factorization (e.g., singular value decomposition), thus uncovering latent node representations [20,21,22]. Although there are several variations, they generally correspond to a structure-preserving dimensionality reduction process. Unlike our method, despite being unsupervised and mathematically transparent, these solutions often ignore semantics and only conserve first-order proximity (local pairwise similarity between nodes linked by edges) [23]. Moreover, either matrix construction or eigendecomposition is time- and space-consuming, making matrix factorization inefficient and unscalable for large graphs [24] (even if this is not the case with events).

Deep Learning. DL has shown promising results among different embedding methods thanks to the automatic identification of suitable representations from complex graph structures as optimization problems. This line of work aims to design objective cost functions that capture intra-graph dependencies and similarities while preserving high quality in downstream tasks and constructing graph embeddings under efficiency constraints. Here, we divide DL contributions into two categories depending on whether the input is formed by sampled random walks (i.e., node sequences) rather than the whole graph.

Deep Learning with Random Walks. Sequence-based embeddings are built on the fundamental idea of linearizing graphs. DeepWalk from Petroni et al. [23] was the first attempt to generalize skip-gram NLP models to graph-structured data, drawing analogies between language and graphs. In this perspective, each path sampled from a graph corresponds to a corpus sentence, where a node equals a word. More in detail, DeepWalk performs multiple random walks from each node and trains neural networks by maximizing the probability of predicting the node’s context (local neighborhood structure) conditioned on the embedding of the node itself, encoding co-occurrences in short sub-windows. Many studies optimize, modify, or extend this idea, with prominent examples in LINE [25], node2vec [26], metapath2vec [27], HARP [28], WYS [29], and GEMSEC [30]. DeepWalk-based methods are unsupervised and can conserve second-order proximity. They capture long-distance relationships and place nearby nodes with similar neighborhoods, even if not directly connected. Sampling allows for exploring and simplifying the graph, lacking awareness of global graph information but keeping computation tractable. Thus, it is proper for single massive networks containing millions of nodes and billions of edges, drawing an opposite scenario from ours. Worst, while there are some exceptions [31], many of these algorithms only work with structure.

Deep Learning without Random Walks. These methods directly apply DL models to entire graphs. One common approach is adopting *autoencoders*, which usually have an encoding and decoding network to model nonlinearities. If we input an adjacency matrix, the encoder aggregates local information at a node level by producing compressed representations, and the decoder minimizes a reconstruction loss from node embeddings (ensuring neighborhood preservation as an unsupervised objective). For example, GAE and VGAE [32] reconstruct the graph structure by taking the dot product between the node embeddings encoded by a graph neural network (GNN). *GNNs* are precisely another methodological family within this class. They aim to learn differentiable functions over graphs with an arbitrary structure for solving supervised (or semi-supervised) tasks, generally requiring a large volume of labeled data to discover meaningful representations. While earlier techniques first map nodes into latent vector representations and then pass them as inputs to another neural network, supervised graph embedding combines these two steps. Hence, according to node feature signals, GNNs learn embeddings for a specific purpose (e.g., predicting molecular properties or chemical compound toxicity) and cannot be used or transferred to other tasks or problems. More concretely, such models are invariant to permutations of graph elements by design and compute graph node representations through a propagation process that iteratively aggregates local information. One of their main advantages is being inductive cause GNNs capture functions applicable to any graph of the supported type and not just those treated as inputs during training. Three highly cited works are graph convolutional networks (GCNs) [33], graph attention networks (GATs) [34], and GraphSage [35]. GCNs replace message passing with graph convolutions. GATs use masked self-attention layers to balance neighbors’ impact during aggregation. GraphSage improves GCNs by sampling only a fixed number of weighted neighboring nodes at different depths, thus integrating random walks. Unlike these methods, DDEGK learns task-agnostic and unsupervised event representations, without being limited to homogeneous and non-relational graphs (type of input traditionally considered by many GNNs).

For more information on this area, we refer the reader to recent surveys [24,36,37]. Node that embeddings alone can be used to calculate inter-graph similarities. For example, in Nikolentzos et al. [38], the nodes of two graphs are projected in the Euclidean space using the eigenvectors of the adjacency matrices, representing each graph as a bag-of-vectors. The similarity is then measured by computing a matching based on the Earth Mover’s Distance [39] between the two sets of embeddings. More originally, node2vec-PCA [40] brings node embeddings into a reduced-dimensional space and uses latent similarities between nodes to encode graphs as 2D histogram stacks; the artificial image thus constructed can then be passed to a classical 2D CNN architecture for supervised tasks. In any case, despite the extensive development of node embedding techniques, a well-known problem concerns the neglect of the global structure and higher-level patterns, which are truly necessary for comparison. Thus, as ordinarily required by small graphs, like proteins and molecules [37], we aim to learn a single representation for each biomedical event graph and not for each event node.

#### 2.1.2. Whole-Graph Embedding

Much effort has focused on node-, edge-, or graph-level supervised learning and node-level unsupervised learning (e.g., node clustering). By contrast, research on *graph-level unsupervised learning* has so far received relatively little attention, despite its wide range of practical applications for graph matching or similarity ranking in domains like biology and NLP [41]. Embedding a whole graph requires choosing between expressive power and efficiency, introducing some notion of pooling to aggregate sub-level information into a single vector. As discussed earlier, generalizing this notion of pooling to arbitrary graphs is non-trivial because of the lack of regularity in the graph structure; thus, it still denotes an active research area.

Supervised learning. Prevalent solutions include (i) simply summing up or averaging all node embeddings in a final layer [42,43], (ii) adding parametric graph pooling layers (e.g., DiffPool [44], SortPool [45], TopKPool [46], SAGPool [47]) or memory layers (e.g., MemGNN [48]) within supervised GNNs. Alternatively, Patchy-San [49] proposes an approach for convolutional operations on graph structured data, with both discrete and continuous node and edge attributes.

Unsupervised learning. Leaving aside GNNs and supervised techniques, which are clearly distinct from ours, some unsupervised methods have been proposed [50,51,52]. The most intuitive way of generating one embedding per graph using a set of node embeddings is applying flat (e.g., mean, max, sum) or hierarchical pooling. One could also perform a weighted sum driven by node degree or node relevance for the graph, detectable by context-aware attention [53] or by term-weighting schemes [54] in case of text labels. Still another possibility is introducing a virtual super-node [41]. More advanced solutions include sub2vec [50] and graph2vec [51]. Sub2vec learns representations of any subgraphs by sampling fixed-length random walk linear substructures; nevertheless, it has disadvantages in terms of instability and information loss. Working with nonlinear substructures, graph2vec better preserves structural equivalence and currently has state-of-the-art performances on several datasets. As node2vec is an analog to word2vec, graph2vec is an analog to doc2vec. In fact, starting from the observation that graphs are sets of subgraphs in the same way documents are sets of words, the authors adapt doc2vec from NLP and train a skip-gram model to predict subgraphs that exist in the input graph. However, graph2vec is transductive, meaning that it only yields an embedding for instances known at training time.

Statistical Representations. Another branch of work [55,56] represents graphs with hand-engineered feature vectors, using them for subsequent inter-graph comparisons. They aggregate local characteristics, statistical, or topological properties (like the degree of a node and its neighbors) and are oblivious to global perspectives, often requiring known node-to-node mappings. In contrast, DDEGK does not explicitly engineer its features and makes no assumptions about their importance for the application task.

Whole-graph embedding provides a straightforward and efficient solution for calculating graph similarities, combining speed and scalability. However, there are also shortcomings: graph-graph proximity is ignored, and there are no feature interactions across graphs. Consequently, models may be unsuitable for graph similarity predictions compared to joint methods integrating GRL with GSL. To solve this problem, DDEGK learns whole-graph embeddings by graph comparisons.

#### 2.1.3. Knowledge Graph Embedding

In recent years, network embedding research has intensely studied also special graphs, like knowledge graphs (KGs) [57]. KGs are symbolic abstractions used for encoding a knowledge base, a collection of statements, often referred to as “facts”, having the form of interlinked *subject-predicate-object* (SPO) triples. Widely adopted in NLP tasks, what makes KGs somewhat special is that they come with labeled nodes as well as labeled edges (or, equivalently, many different binary relations). Since accommodating the methods of Section 2.1.1 and Section 2.1.2 to this environment is not trivial, KG embedding has been notably addressed by a community that seems detached from the one described so far. Most current methods create a graph-coherent vector for each entity and relation (i.e., hybrid embedding), with a learning process centered on distinguishing correct triples from negative (corrupted [58]) ones. Peculiarly, translational (or “fact alone”) models only exploit the triplet structure to learn head→tail translations (e.g., TransE [59] and its evolutions [57]). Instead, bilinear models use a multiplicative approach to represent relationships as matrices in the vector space (e.g., DistMult [60], HolE [61], and ComplEx [62]). Ultimately, we have neural models, such as neural tensor networks (NTNs) [63], RDF2Vec [64], ConvE [65], and R-GCNs [66]. Some researchers further incorporate additional information like textual descriptions to semantically enrich KG representations [67,68,69]. While there is a large body of work on embedding KGs, that is, binary relational structures, not much is known about embedding relations of higher arities [70], like events. Obviously, a reasonable approach to embed n-ary connections is to break them down into binary incidence structures (set of pairwise edges). On the flip side, events express complex concept units, and we note that single triples within them can originate incomplete or incorrect facts. Moreover, events directly associate trigger-entity or trigger–trigger pairs, but not entity–entity ones. Accordingly, unlike these works, our solution faces the problem of computing embedding for independent n-ary relational facts instead of edges contained into a single large source graph.

### 2.2. Graph Similarity Computation with Cross-Graph Feature Interaction

The following methods take a pair of graphs as input and compute a similarity score between them. Compared to those described in Section 2.1, the matching occurs jointly on the pair, rather than independently mapping each graph to a vector. Therefore, these models are potentially stronger than the standard embedding models at the cost of some extra computation. We also emphasize that, in many problem domains, it is easier to specify a reasonable dissimilarity (or similarity) function between instances rather than to construct a feature representation of the whole structured input [71].

#### 2.2.1. Direct Comparison Methods

Early on, multiple graph similarity metrics were proposed based on predefined and concrete distance notions, like the Graph Edit Distance (GED) [72] or the size of the Maximum Common Subgraph [73]. Unfortunately, the computation of these metrics is NP-Complete in the general case, implying exponential time complexities which can be unsustainable even for instances having more than 16 nodes [14]. Given the great difficulty of computing exact graph distances, pruning strategies [74] and heuristic methods [75] are regularly used to reduce similarity computation to a tractable degree. In addition, GED treats all edit operations as equal, without discerning the extent to which they may alter the graph topology or semantics, remaining unsatisfactory for comparing different-size graphs. Unlike these approaches, our method deliberately avoids algorithmic insights.

#### 2.2.2. GNN-Based Graph Similarity Learning

This family of GSL techniques uses GNNs to simultaneously learn both graph representations and graph similarities in an end-to-end fashion. Given pairs of input graphs $\langle G_{i},G_{j},y_{ij}\rangle$, where $y_{ij}$ denotes the ground-truth similarity score of $\langle G_{i},G_{j}\rangle$, such techniques first employ multi-layer GNNs to learn the embeddings of $G_{i}$ and $G_{j}$, where each graph could influence the other by some mechanisms like weight sharing and cross-graph interactions. The prediction of the similarity score between the two graph vector or matrix representations returned by the GNNs is computed using a dot product layer or fully connected layers. Similarity estimates for all graph pairs and ground-truth labels are finally compared within a loss function for gradient updates.

GNN-CNN. GNN-CNN mixed networks (e.g., GSimCNN [76], SimGNN [53]) adopt GNNs to learn graph representations that are then exploited into CNNs for predicting similarity scores, casting the problem to classification or regression.

Siamese GNN. Siamese GNN models (e.g., S-GCN [77], HS-GCN [78], MatchGNet [79], UGRAPHEMB [80]) consist of twin GNNs with shared parameters, independently applied to two input graphs to produce graph representations then fused by a small network for similarity prediction. Finally, the similarity estimate is leveraged in a loss function for training the entire model.

Graph Matching Networks. Works in this category (e.g., GMN [17]) adapt Siamese GNNs by incorporating matching mechanisms and cross-graph interactions during the GRL process conducted by the two GNNs.

Many graph-pair distance scores need to be labeled in advance for training all these methods. Instead, we use no labels about similarity scores, prompted by the absence of datasets in this regard for biomedical events and events in general.

#### 2.2.3. Graph Kernels

Graph kernels evaluate the similarity (kernel value) between graph pairs by recursively decomposing them into atomic substructures over which define a similarity function (kernel). Traditional graph kernels use handcrafted and graph-theory-motivated features, including random walks, shortest paths, graphlets, etc. For instance, shortest-path kernels [81] compare the lengths of all shortest paths between vertices in two graphs. Graphlet kernels [82] count the number of {3,4}-sized subgraphs (motifs). Weisfeiler–Lehman kernels [83] propose to aggregate discrete or continuous information on nodes via the analog of the color refinement heuristic for isomorphism testing. Multi-scale Laplacian kernels [84] compare graphs at different scales, detecting topological relationships between nodes and subgraph associations. However, explicitly defined kernels are hampered by problems like high dimensional, sparse, and non-smooth representations, thus yielding to poor generalization [85]. Deep graph kernel models have recently emerged, replacing manually designed features with ones learned automatically from data via deep neural networks. According to a recent GSL survey [15], DDGK [19] is the only contribution capable of managing heterogeneous and attributed graphs, supporting cross-graph interactions, with already tested applications on chemoinformatics and bioinformatics. Our work still belongs to this group, but focuses on event embeddings.

### 2.3. Event Embedding

Events are key for revealing underlying real-world knowledge and capturing biological processes described in the unstructured text. Unfortunately, many contributions on event extraction [2] are contrasted by a tiny number of works on event representation learning, which constitutes a pressing need. Event embedding is an efficient solution to represent discrete and sparse events as dense vectors in a continuous space, reflecting their similarity and availing numerous applications. The critical problem that unites almost all the current event embedding methods is the consideration of binary relations only [86,87,88,89,90]. This simplification portrays an unrealistic case, far from the complex concept units targeted in popular competitions like BioNLP-STs and ACE2005. Given the predominant role of semantics in events, many of these methods [86,87,88] see events as SPO tuples and employ NTNs typically used for KG embedding. With NTNs, the authors learn interactions between the predicate and its subject/object, generating node/edge embeddings, using corrupted tuples for training, and eventually incorporating external knowledge [87,89]. Karumba [90] explores the supervised learning of events with hierarchical structures in hyperbolic space. DeepEventMine [91], the current state-of-the-art solution for end-to-end biomedical event extraction, internally adopts event representations mainly composed of BERT-based embedding concatenations, essentially ignoring structure. Remarkably, Gui et al. [92] do not decompose the interaction among all participating entities into several independent and scattered pairwise relations, but employ an hyperedge structure specifically to avoid information loss. By the same principle, we encode multiple interactions as a whole. Our work poses, to the best of our knowledge, the first method for graph-level event embedding based on deep graph kernels.

## 3. Notation and Preliminaries

Before continuing with our contribution, we formally provide the necessary notation and definitions of the core concepts that will be used throughout the paper.

**Definition 1**. *(Event Graph). A graph, denoted by G=(V,E), consists of a finite set of vertices, $V=v_{1},\ldots ,v_{\left|V\right|}$, and a set of edges, E⊆V×V, |E|=m, where an edge $e_{i,j}$ connects vertex $v_{i}$ to vertex $v_{j}$. In the case of event graphs, edges are directed, unweighted, and there are no cycles. A vertex represents a trigger or an entity, while an edge models an entity-trigger or a trigger–trigger relation, with the second applying for nested events. Node connections are encoded in an adjacency matrix $A\in R^{\left|V|\times |V\right|}$, where $a_{ij}=1$ if there is a link between nodes i and j, and 0 otherwise. Nodes and edges in G are associated with type information. Let $\tau_{v}:V\to T^{v}$ be a node-type mapping function and $\tau_{e}:E\to T^{e}$ be an edge-type mapping function, where $T^{v}$ indicates the set of node (event or entity) types, and $T^{e}$ the set of edge (argument role) types. Each node $v_{i}\in V$ has one specific type, $\tau_{v}\left(v_{i}\right)\in T^{v}$; similarly, for each edge $e_{ij}$, $\tau_{e}\left(e_{ij}\right)\in T^{e}$. Since $|T^{v}\left|+\right|T^{e}|>2$, event graphs are heterogeneous networks. An event graph is also endowed with a label function $\gamma_{v}:V\to \Gamma^{v}$ that assigns unconstrained textual information to all nodes. We say that $\gamma_{v}\left(v_{i}\right)$ is the continuous attribute of $v_{i}$.*

**Definition 2**. *(Graph and Subgraph Isomorphism). Two unlabeled graphs $G_{1}$ and $G_{2}$ are isomorphic, denoted by $G_{1}≃G_{2}$, if there exists a bijection $\phi :V\left(G_{1}\right)\to V\left(G_{2}\right)$, such that $\left(u,v\right)\in E\left(G_{1}\right)$ if $\left(\phi \left(u\right),\phi \left(v\right)\right)\in E\left(G_{2}\right)$ for all (u,v) in $E(G_{1})$. Then, ϕ is an isomorphism. For labeled graphs, isomorphism holds only if the bijection maps vertices and edges with the same label. Subgraph isomorphism is a generalization of the graph isomorphism problem, where the goal is to determine whether $G_{1}$ contains a subgraph that is isomorphic to $G_{2}$. No polynomial-time algorithm is known for graph isomorphism. Accurately, while subgraph isomorphism is known to be NP-complete, the same cannot be said for graph isomorphism, which remains NP.*

**Definition 3**. *(Graph Kernel). Given two vectors x and y in some feature space $R^{n}$, and a mapping $\varphi :R^{n}\to R^{m}$, a kernel function is defined as $k\left(x,y\right)=\varphi {\left(x\right)}^{T}\varphi \left(y\right)$. In a nutshell, a kernel is a similarity function—satisfying the conditions of symmetry and positive definiteness—that can be interpreted as the dot product of two vectors after being projected into a new space. Thus, a kernel function can be applied to compute the dot product of two vectors in a target feature space (typically high-dimensional) without the need to find an explicit space mapping. In structure mining, a graph kernel is simply a kernel function that computes an inner product on graph pairs, typically comparing local substructures [93].*

**Definition 4**. *(Whole Graph Representation Learning). Whole graph representation learning aims to find a mapping function Ψ from a discrete graph G to a continuous vector $\Psi \left(G\right)\in R^{d}$, preserving important graph properties. If d is low, we talk about graph embedding. Graph embedding can be viewed as a dimensionality reduction technique for graph-structured data, where the input is defined on a non-Euclidean, high-dimensional, and discrete domain.*

**Definition 5**. *(Graph Similarity Learning). Let G be an input set of graphs, $G=G_{1},G_{2},\ldots ,G_{n}$, graph similarity learning aims to find a function $S:(G_{i},G_{j})\to R$, returning a similarity score $s_{ij}$ for any pair of graphs $(G_{i},G_{j})\in G$.*

## 4. Materials and Methods

### 4.1. Datasets

Nine real-world datasets—originally designed for biomedical event extraction—are used within this paper to evaluate DDEGK on gold instances (and not silver ones), thus avoiding additional error sources in system predictions. Most of them are benchmarks with human-curated labels introduced by the ongoing BioNLP-ST series, one of the most popular community efforts in biomedical text mining. Each deals with topics from a distinct sub-area of biology, supported by literature-based corpora.

- *BioNLP-ST 2009 (ST09)* [4]. Dataset taken from the first BioNLP-ST challenge, consisting of a sub-portion of the GENIA event corpus. It includes 13,623 events (total between train, validation, and test sets) mentioned in 1210 MEDLINE abstracts on human blood cells and transcription factors.
- *Genia Event 2011 (GE11)* [94]. Extended version of ST09, also including ≈4500 events collected from 14 PMC full-text articles.
- *Epigenetics and Post-translational Modifications* (EPI11) [95]. Dataset on epigenetic change and common protein post-translational modifications. It contains 3714 events extracted from 1200 abstracts.
- *Infectious Diseases (ID11)* [96]. Dataset on two-component regulatory systems; 4150 events recognized in 30 full papers.
- *Multi-Level Event Extraction (MLEE)* [3]. Dataset on blood vessel development from the subcellular to the whole organism; 6667 events from 262 abstracts.
- *Genia Event 2013 (GE13)* [97]. Updated version of GE11, with 9364 events extracted exclusively from 30 full papers.
- *Cancer Genetics (CG13)* [98]. Dataset on cancer biology, with 17,248 events from 600 abstracts.
- *Pathway Curation (PC13)* [99]. Dataset on reactions, pathways, and curation; 12,125 events from 525 abstracts.
- *Gene Regulation Ontology (GRO13)* [100]. Dataset on human gene regulation and transcription; 5241 events from 300 abstracts.

#### 4.1.1. Data Preprocessing and Sampling

**Figure 5:**
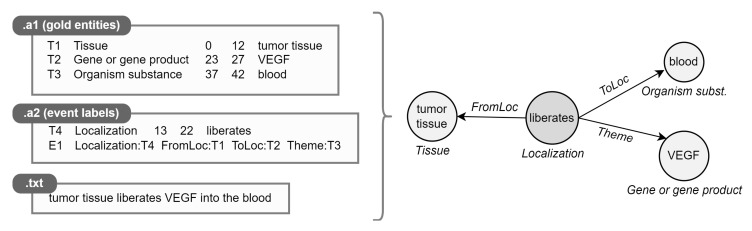
Example of .a* parsing. The textual document is shown for clarity.

Instances within the datasets are formed by a text document (*.txt*) and two annotation files in a standard format adopted by BioNLP-ST, one dedicated to gold entities (*.a1*) and one including triggers with their relationships (*.a2*). We parse standoff files (.a*) to automatically transform labeled text spans into event graphs (Figure 5).

**Figure A1:**
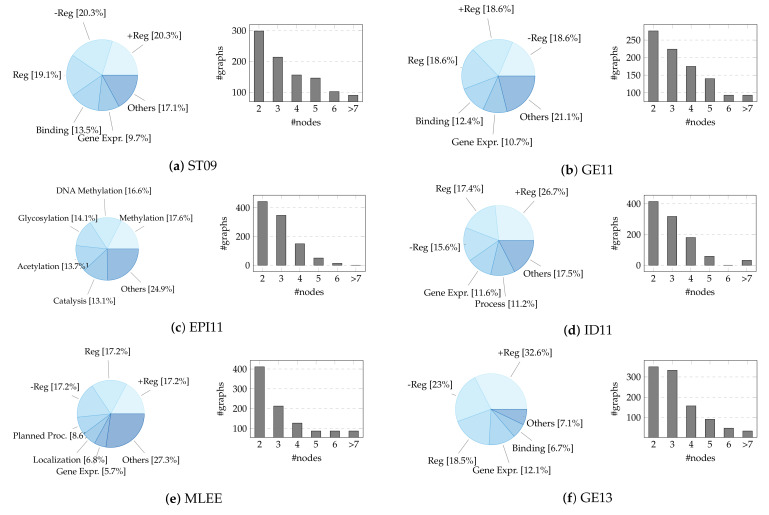

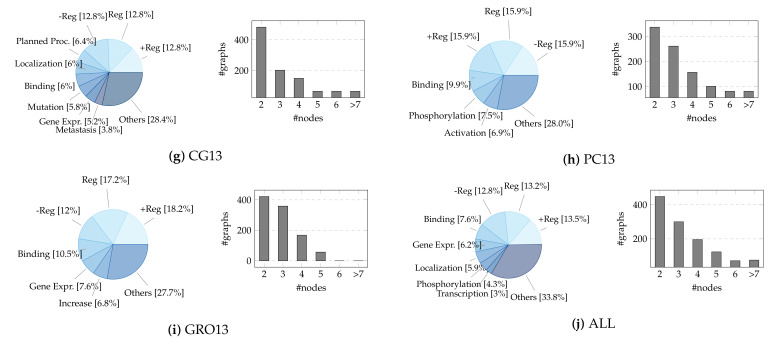
Distribution of event types (root ones, in case of nesting) and graph sizes within sampled datasets.

Given the large number of records, we build a sample of ≈1000 events from the training set of each dataset using stratified random sampling (without replacement) to retain the statistical information of the population. We stratify on multiple variables, namely the event type (the main one in case of nesting, i.e., the event graph root) and the node number (discretized in a range distribution, i.e., 2, 3, 4, 5, 6, >7), keeping their original proportions. We remove event types represented by less than ten instances due to highly unbalanced data. To experiment with mapping events from different sources towards a single shared space, we create an artificial dataset—hereafter referred to as “bio_all”—resulting from the combination of the previous nine (additionally stratifying on the source). The selected sample dimension is comparable to or greater than that of other chemo/bio-informatics graph classification datasets, like D&D [101] (1178), PTC-MR [102] (344), ENZYMES [103] (600), and MUTAG [104] (188). The reader should be aware that the test sets of the BioNLP-ST corpora are generally not provided, leading users to upload their predictions to the task organizers’ servers. Due to the unavailability of these data, we refer only to the event instances included in the training sets. A concise summary and detailed visualization of the final datasets can be found in Table 1 and Figure A1, respectively. The restricted size of event graphs is offset by the high diversity of classes (17, on average, compared to the 2–6 labels of conventional biomedical GRL benchmarks [105]).

### 4.2. Deep Divergence Event Graph Kernels

This section describes how DDEGK works. It recalls the original DDGK method and highlights our changes, including a new loss function and specific variations to handle biomedical events.

**Table 1:** Descriptive statistics for sampled datasets. The first column lists the examined datasets. The second column indicates the number of event graph instances (nested events count one), while the third and fourth macro-columns detail the size of events in terms of nodes and edges. Finally, the last macro-column refers to the distinct number of types for events (triggers), entities, and argument roles in each dataset, thus marking the number of possible classes for graphs, nodes, and edges.

Dataset	# Graphs	# Nodes			# Edges	# Labels		
		Min	Mean	Max	Mean	Graph	Node	Edge
ST09	1007	2	4	14	3	9	11	3
GE11	1001	2	4	14	3	9	11	2
EPI11	1002	2	3	6	2	10	11	1
ID11	1001	2	3	14	2	9	16	3
MLEE	1012	2	4	15	3	15	39	8
GE13	1011	2	3	13	2	8	13	2
CG13	1033	2	3	13	2	23	50	8
PC13	1020	2	4	18	3	15	25	9
GRO13	1006	2	3	5	2	18	140	4
BIO_ALL	1216	2	3	14	3	53	101	12

#### 4.2.1. Problem Definition

Given a family of *N* event graphs $T=G_{1},G_{2},\ldots ,G_{N}$, our goal is building an embedding for each event graph $G_{i}$ (which we call *target* event graph), based on its distance from a set of *M anchor (or source)* event graphs (A). To accomplish this, we aim to learn a graph kernel function, defined as the following:

(1) $$k\left(G_{1},G_{2}\right)=||\Psi \left(G_{1}\right)-\Psi \left(G_{2}\right){\left|\right|}^{2},$$

where the representation $\Psi \left(G\in T\right)\in R^{M}$. In particular, for any member of T, we define the $i^{\mathrm{th}}$ dimension of the representation to be:

(2) $$\Psi {\left(G\right)}_{i}=\sum_{v_{j}\in V\left(G\right)}f_{a_{i}}\left(v_{j}\right),$$

where $a_{i}\in A$ and $f_{a_{i}}\left(\right)$ is a predictor of some structural and semantic properties of the graph *G* parameterized by the graph $a_{i}$. The anchor and target graphs sets (A, T) could be disjoint, overlapping, or equal.

**Figure 6:**
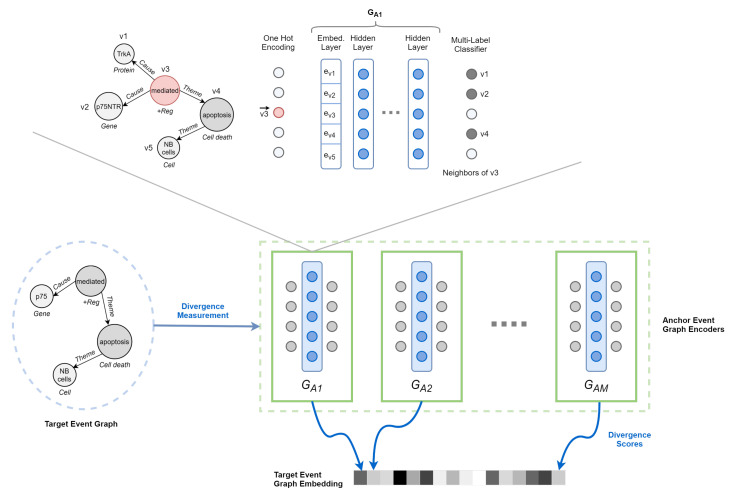
Overview of DDEGK. Structural and semantic divergence scores from a set of anchor event graphs are used to compose the vector representation of a target event graph. Divergence is measured through pre-trained Node-to-Edges encoder models, one for each anchor.

In other words, we propose to learn the representation of an event graph by comparing it against a population of other event graphs taken as a reference and forming the basis of our target vector space (Figure 6).

#### 4.2.2. Event Graph Representation Alignment

To define the similarity between two event graphs in a $\langle target,anchor\rangle$ pair Equation (2), we rely on deep neural networks. We train an encoder model to learn the structure of the anchor event graph. The resulting model is then used to predict the structure of the target event, allowing divergence measurement. If the pair is similar, it is natural to expect the anchor encoder to correctly predict the structure of the target event graph. However, two event graphs may not share vertex ids and differ in size, albeit similar in the concept unit expressed. Likewise, nodes of structurally equivalent graphs can have completely different attributes. For this reason, we use a cross-graph attention mechanism to learn a soft alignment between the nodes of the target event graph and the anchor one.

Below, we illustrate the functioning of the various components.

**Anchor Event Graph Encoder**. The quality of the graph representation depends on the extent to which each encoder is able to discover the structure of its anchor. Therefore, the role of the encoder is to reconstruct such structure given partial or distorted information. Analogously to Al-Rfou et al. [19], we choose a Node-To-Edges setup, where the encoder is trained to predict the neighbors of a single vertex in input. Although other strategies are undoubtedly possible, it greatly matches event graph characteristics while remaining efficient and straightforward to process. By modeling the problem as a multi-label classification task, we maximize the following objective function:

(3) $$J\left(\theta \right)=\sum_{i}\sum_{\begin{array}{c} j\\ e_{ij}\in E \end{array}}logPr\left(v_{j}|v_{i},\theta \right).$$

First, each vertex $v_{i}$ in the graph is represented by a one-hot encoding vector ${\vec{v}}_{i}$. Second, we multiply the encoding vector with a linear layer $E\in R^{|V|\times d}$, resulting in an embedded vertex $e_{v_{i}}\in R^{d}$, where *d* is the size of the embedding space. Third, the embedding $e_{v_{i}}$ (feature set) thus obtained is passed to a fully connected deep neural network (DNN), which produces scores for each vertex in *V* (i.e., output layer of size |V|). Finally, scores are normalized using the sigmoid function to generate final predictions.

**Cross-Graph Attention**. To compare pairs of event graphs that may differ in size (node sets) and structure (edge sets), we need to learn an alignment between them. For this to happen, we employ an attention mechanism encoding a relaxed notion of graph isomorphism. This *isomorphism attention* bidirectionally aligns the nodes of a target graph against those of an anchor graph, ideally operating in the absence of a direct node mapping and drawing not necessarily one-to-one correspondences. It requires two separate attention networks. The first network, denoted as $M_{T\to A}$, allows nodes in the target graph ($G_{T}\in T$) to attend to the most structurally similar nodes in the anchor graph ($G_{A}\in A$). Specifically, it assigns every node $t_{i}\in V\left(G_{T}\right)$ a probability distribution (softmax function) over the nodes $a_{j}\in V\left(G_{A}\right)$, i.e., 1:N mapping. On the implementation level, we use a multiclass classifier:

(4) $$Pr\left(a_{j}|t_{i}\right)=\frac{e^{M_{T\to A}\left(t_{i},a_{j}\right)}}{\sum_{a_{k}\in V\left(G_{A}\right)}e^{M_{T\to A}\left(t_{i},a_{k}\right)}}$$

The second network, denoted as $M_{A\to T}$, is a reverse attention network which maps a neighborhood in the anchor graph to a neighborhood in the target graph, i.e., N:N mapping. We implement it as a multi-label classifier with a sigmoid function:

(5) $$Pr\left(t_{j}|N\left(a_{i}\right)\right)=\frac{1}{1+e^{-M_{A\to T}\left(N\left(a_{i}\right),t_{j}\right)}}$$

By wrapping the anchor event graph encoder with both attention networks, we are able to predict the neighbors of each node within a target graph—but utilizing the structure of the source graph. Such isomorphism attention allows for capturing higher-order structure between graphs, going beyond the immediate neighborhoods.

**Attributes Consistency**. As we know, event graphs are not defined only by their structure but also by the attributes of their nodes and edges. Accordingly, to learn an alignment that preserves semantics, we add regularizing losses to the attention and reverse-attention networks. Compared to DDGK, we add support for continuous attributes, indispensable in the event domain.

As for the node type $\tau_{v}\left(\right)$, we estimate a probability distribution of the discrete attributes over the target graph based on the learned attention scores, defined as follows:

(6) $$Q_{\tau_{v}}\left(y_{i}|t_{j}\right)=\sum_{k}M_{T\to A}\left(y_{i}|a_{k}\right)Pr\left(a_{k}|t_{j}\right),$$

where $M_{T\to A}\left(y_{i}|a_{k}\right)$ simply checks whether the predicted anchor nodes have label $y_{i}$ or not (1 or 0). To define the attention regularizing loss over the node types, we adopt the average cross-entropy loss with the aim of measuring the difference between the observed distributions of discrete attributes $Pr(y_{i}|t_{j})$ and the inferred ones $Q_{\tau_{v}}\left(y_{i}|t_{j}\right)$:

(7) $$L_{\tau_{v}}=\frac{1}{\left|V(G_{T})\right|}\sum_{j}^{\left|V(G_{T})\right|}\sum_{i}Pr\left(y_{i}|t_{j}\right)log\left(Q_{\tau_{v}}\left(y_{i}|t_{j}\right)\right)$$

As for the edge type $\tau_{e}\left(\right)$, we set $Q_{\tau_{e}}\left(y_{i}|t_{j}\right)$ to the normalized attributes count over all edges connected to node $t_{j}$. For instance, if a node $t_{j}$ has three edges with two of them labeled as “Theme” and the other as “Cause”, $Q_{\tau_{e}}\left(Theme|t_{j}\right)=0.67$. By replacing $Q_{\tau_{v}}$ with $Q_{\tau_{e}}$ in Equations (6) and (7), we create a regularization loss for edge discrete attributes.

As for the node text $\gamma_{v}\left(\right)$, we minimize the following loss function, as a mean reduction of the cosine distance between the embedding of the textual labels of the nodes in the target and anchor graphs, weighted by their alignment score:

(8) $$L_{\gamma_{v}}=\frac{1}{|V\left(G_{T}\right)|\times |V\left(G_{A}\right)|}\sum_{j}^{\left|V(G_{T})\right|}\sum_{i}^{\left|V(G_{A})\right|}cos\_dist\left(emb\left(\gamma_{v}\left(t_{j}\right)\right),emb\left(\gamma_{v}\left(a_{i}\right)\right)\right)Pr\left(a_{i}|t_{j}\right).$$

In this study, we use SciBERT’s pre-trained embeddings [106]. The key idea is to exploit the linguistic and domain knowledge learned by large biomedical language models, otherwise not included in the event graph.

Inspired by DeepEventMine (state-of-the-art in biomedical event extraction) [91], we implement the emb() function with the subsequent representation $m_{f,l}$, indicating a text span from the first word *f* to the last one *l*:

(9) $$m_{f,l}=\left[v_{f,1};\frac{\sum_{i=f}^{l}\sum_{j=1}^{s_{i}}v_{i,j}}{\sum_{i=f}^{l}s_{i}};v_{l,s_{l}}\right],$$

where [;;] denotes concatenation, while $v_{i,j}$ denotes the *j*th sub-word representation in the *i*th word. More specifically, $v_{f,1}$ is the first sub-word representation of the *f*th word, while $v_{l,s_{l}}$ is the last sub-word representation of the *l*th word.

The presented regularization losses are also introduced for reverse attention networks. In this case, the distribution of attributes refers to the node’s neighborhood, and the node’s neighborhood edges are those appearing at 2-hops distance from the node.

**Figure 7:**
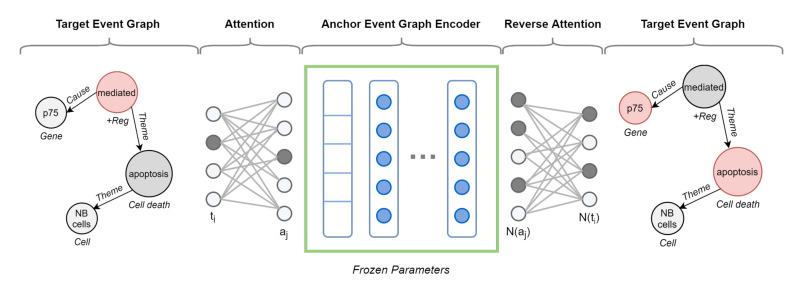
Anchor-based target event graph encoder for divergence prediction. Attention layers map the target event graph nodes onto the anchor graph, being aware of node and edge attributes. The first attention network ($M_{T\to A}$) receives a one-hot encoding vector representing a node ($t_{i}$) in the target graph and maps it onto the most structurally and semantically similar node ($a_{j}$) in the anchor graph. The anchor event graph encoder, then predicts the neighbors of $a_{j}$, $N(a_{j})$. Finally, the reverse attention network ($M_{A\to T}$) takes $N(a_{j})$ and maps them to the neighbors of $t_{i}$, $N(t_{i})$.

Figure 7 shows a vivid example of augmented target graph encoder with cross-graph attention.

#### 4.2.3. Event Graph Divergence and Embedding

Here, we propose using the augmented encoder to define a measure of dissimilarity between a target event graph and an anchor one, from which derive a graph kernel function (Equations (1) and (2)). In other words, the distance measure is learned from the data (distance metric learning) in an unsupervised way and then converted into a kernel.

We note that learning a metric by measuring the divergence score between a pair of graphs $G_{A}$ and $G_{T}$ is possible. We refer to the encoder trained on an anchor graph $G_{A}$ as $H_{G_{A}}$ and the divergence score given to the target graph $G_{T}$ as:

(10) $$D^{\prime }(G_{T}\left|\right|G_{A})\;=\sum_{v_{i}\in V\left(G_{T}\right)}\sum_{\begin{array}{c} j\\ e_{ij}\in E\left(G_{T}\right) \end{array}}-logPr\left(vj|vi,H_{G_{A}}\right).$$

This operation is equivalent to multiplying the probabilities of correct prediction of the neighbors of each target node using the anchor event graph encoder wrapped with semantic and structural alignments. If two graphs are semantically and structurally similar, we expect their divergence to be correspondingly low. Given that $H_{G_{A}}$ is not a perfect predictor of $G_{A}$ structure, we can safely assume that $D^{\prime }(G_{A}\left|\right|G_{A})\neq 0$. To rectify this problem and ensure identity, we define:

(11) $$D^{\prime }(G_{A}\left|\right|G_{T})\;=D^{\prime }(G_{A}\left|\right|G_{T})-D^{\prime }(G_{A}\left|\right|G_{A}),$$

which sets $D(G_{A}||G_{A})$ to zero. However, this definition is not symmetric since $D(G_{T}||G_{A})$ might not necessarily equal to $D(G_{A}||G_{T})$. If symmetry is required, we can use:

(12) $$D\left(G_{A},G_{T}\right)=D(G_{A}\left|\right|G_{T})+D(G_{T}\left|\right|G_{A}).$$

A recent work, called D2KE (distances to kernels and embeddings) [71], proposes a general methodology for deriving a positive-definite kernel from any given distance function d:G×G→R. We adopt D2KE to develop an unsupervised neural network model for learning graph-level embeddings based on the architecture illustrated so far. We select a subset of training samples as a held-out representative set and use distances to points in the set as the feature function. Definitely, we establish a vector space where each dimension corresponds to one event graph in the anchor set. Target event graphs are represented as points in this vector space:

(13) $$\Psi \left(G_{T}\right)=[D(G_{T}\left|\right|G_{A_{0}}),D(G_{T}\left|\right|G_{A_{1}}),...,D(G_{T}\left|\right|G_{A_{M}})].$$

To create a kernel out of our event graph embeddings, we use the Euclidean distance measure as outlined in Equation (2). In essence, our graph kernel identifies topological and semantic associations between individual event nodes.

#### 4.2.4. Training

We extend the original implementation using TensorFlow and Adam optimizer. Expressly, we train *M* anchor graph encoders, then we freeze the parameters and add the two attention networks for target↔anchor mapping and the regularizing losses to preserve semantics. Finally, the augmented encoders are trained on each target event graph in input, represented as an adjacency matrix accompanied by an additional matrix for each attribute type ($\tau_{v}$, $\tau_{e}$, and $\gamma_{v}$).

#### 4.2.5. Scalability

As typical of kernel approaches, our method relies on pairwise similarity and requires N×M computations for scoring each of the *N* target event graphs against each of the *M* anchor event graphs (i.e., quadratic time complexity in terms of the number and size of the graphs). However, in Section 5, we demonstrate that it is not necessary to compute the entire graph kernel matrix to achieve high performance. Best results were generally obtained using only 64 anchors (embedding dimensions) out of a population with more than 1000 biomedical events. Resuming Al-Rfou’s work [19], the total computation cost for training the anchor graph encoders and the attention-augmented target ones can be approximated as $\Theta (N\times M\times T\times \left(V\times d+k\times d^{2}\right))$, where *M* can be much lower than *N*. In this formulation, *T* is the maximum between the encoding and scoring epochs, *V* is the average number of nodes, *d* is the embedding and hidden layer size, and *k* is the maximum between the number of encoder hidden layers and attention ones.

The embeddings of event graphs in a large database can be precomputed and indexed, enabling efficient retrieval with fast nearest neighbor search data structures like locality sensitive hashing [107].

If graph populations were to change over time, for example, new event graphs expressed in the biomedical literature, it would not be necessary to re-calculate the embeddings but only to estimate the divergence between the latest events and the anchor ones (using the previously trained encoders).

### 4.3. Hardware and Software Setup

All the experiments were conducted on a server having a Titan Xp GPU with 12 GB of dedicated memory, 4 CPU cores (Intel i5-6400 2.70 GHz processor), 24 GB of RAM, and running Ubuntu 16.04.6 LTS. For minor tasks and tests, we moved on Google Colab.

## 5. Experiments

Once the event embeddings are computed using DDEGK, they could be leveraged for numerous downstream graph analytics tasks, such as classification, clustering, and similarity search. In this section, we perform a series of quantitative and qualitative experiments to demonstrate the effectiveness of our method compared to the others. First, we show that event graph embeddings learned by DDEGK represent a sufficient feature set to predict the biological area and fine-grained type of a large number of scientific interactions. Then, we prove that representations of events similar in structure and semantics are correctly grouped, assessing the geometrical quality of the kernel space through clustering indices and visualization techniques. Moreover, we present the advantage of event representations over traditional sentence embeddings in the semantic textual similarity (STS) task. Finally, we underline the benefits of cross-graph attention for interpretability purposes.

### 5.1. Event Graph Classification

Our learned event representations respect both structure and discrete/continuous attributes. So, they can be used for graph classification tasks where the graph structure, node attributes, and edge attributes convey meaning or function. To demonstrate this, we use DDEGK representations of the event datasets presented in Section 4.1 as features for graph label predictions, learning a mapping function between event embeddings and classes. Specifically, we evaluate the meaningfulness of the learned representations with two classification tasks: (i) predicting the principal event type of each graph and, therefore, the nature of the expressed interaction; (ii) predicting the biological area of each graph, which is roughly represented by the belonging dataset. The second point refers to the “bio_all” shared space (events with heterogeneous datasets).

#### 5.1.1. Baseline Methods

We compare the performance of DDEGK against a variety of graph embedding models—both supervised and unsupervised—that preserve different event graph properties. These models are representative of the main types of embedding techniques and can be categorized into four groups.

- *Node embedding flat pooling*. Each event is represented as the unsupervised aggregation of its constituent node vectors. We experiment with multiple unweighted flat pooling strategies, namely, mean, sum, and max. As for node representations, we examine (i) contextualized word embeddings from large-scale language models pre-trained on scientific and biomedical texts, (ii) node2vec [26]. The first point is realized by applying SciBERT [106] and BioBERT [108] (768 embedding size) on trigger and entity text spans: a common approach in the event-GRL area [88]. It condenses the semantic gist of an event based on the involved entities; it totally ignores structure and argument roles. In contrast, node2vec is a baseline for sequential methods which efficiently trade off between different proximity levels. The default walk length is 80, the number of walks per node is 10, return and in-out hyper-parameters are 1, and embedding size is 128.
- *Node embedding + CNN*. We use node2vec-PCA [40] (d = 2, i.e., one channel), which composes graph matrices from node2vec and then applies a CNN for supervised classification.
- *Whole-graph embedding*. We use graph2vec [51] to generate unsupervised structure-aware graph-level representations for our biomedical events. We work on labeled graphs, with labels denoting numerical identifiers for event types and entities. Default embedding size is 128, and the number of epochs is 100.
- *GNN + supervised pooling*. We use DGCNN [45], an end-to-end graph classification model made by GCNs with a sort pooling layer to derive permutation invariant graph embeddings. 1D-CNN then extracts features along with a fully-connected layer. Default k (normalized graph size) is 35.

During the experiments, we used the default hyper-parameter setting suggested by the authors and detailed above.

#### 5.1.2. Hyperparameters Search

We first obtain the embeddings of all the event graphs and then feed them to SVM as a kernel classifier (except for DGCNN since it is a supervised algorithm). We split event samples into train, validation, and test sets for overfitting avoidance. To choose DDEGK hyperparameters (Table 2), we perform grid searches for each dataset. At this juncture, we empirically note that, on the datasets under examination, the best results on average are obtained with a semantic-structure ratio of about 20:1, weighting the contribution of each label equally. For SVM, we use the scikit-learn implementation and 10-fold cross validation. We vary the kernel between {linear,rbf,poly,sigmoid} and the regularization coefficient *C* between 10 and $10^{9}$. We choose the combination of DDEGK and classifier hyperparameters that maximize the accuracy on the dev set. Furthermore, we experiment with two different anchor graph choices: (i) a random subset of the original graph set, and (ii) a version balanced with respect to the event types. The second is meant to produce more interpretable vectors, where each event is represented as a mixture of its types (similar to topic modeling [109]). To keep computation tractable, tested dimensions during sampling are 32, 64, and 128.

#### 5.1.3. Results

Graph classification results are shown in Table 3. We measure the test’s accuracy with the F1-score, i.e., the harmonic mean of precision and recall (Equation (14)):

(14) $$F_{1}=2\times \frac{\mathrm{precision}\times \mathrm{recall}}{\mathrm{precision}+\mathrm{recall}}$$

We see that DDEGK performs surprisingly well for an unsupervised method with no engineered features. Our model significantly outperforms the baselines on principal event type classification tasks, achieving higher average accuracy in each scenario (i.e., absolute margins of 22.46 and 6.86 over the best unsupervised and supervised baseline, respectively). For dataset (biomedical area) classification, it also achieves competitive results, only being exceeded by DGCNN (supervised). The performance gap between purely semantic and structural approaches (such as pooling on word embedding compared to node2vec and node2vec-PCA) clearly exhibits the pivotal role of node/edge labels within event modeling. We perceive graph2vec as a compromise between the two. Therefore, the use of auxiliary information seems to be the main driver of classification quality. The average on the embeddings generated by BERT-based models turns out to be the most effective pooling solution, with SciBERT slightly more solid than BioBERT. Considering both node connectivity and node/edge semantics, the deep gap between our solution and SciBERT AVG confirms the validity of a hybrid embedding strategy. Performance tends to decrease in proportion to graph size and label variety. Expectedly, node2vec proves to be highly ineffective on GRO13 due to the low structural variance and limited contextual information. The partial annotation overlap between datasets [110] justifies the lower scores within event spaces having heterogeneous sources. The two strategies for choosing anchor events are comparable in terms of performance.

### 5.2. Between-Graph Clustering

Graph clustering is particularly useful for discovering communities and has many practical applications, such as grouping proteins with similar properties. In our context, it allows the automatic identification of similar events mentioned in the biomedical literature, categorizing them and allowing their quantification (e.g., how many times a specific protein-symptom interaction has been reported). We compare graph embedding methods on biomedical events by examining their clustering quality to understand the global structure of the encoding spaces quantitatively. Exactly, we verify the close location in the vector space of instances sharing the same type or biomedical area of origin. Since we treat whole-graph embeddings, we perform between-graph clustering: starting from a large number of graphs, we attempt to cluster them (not their components) based on underlying structural and semantic behaviors. In contrast with the conventional within-graph clustering, where the goal is partitioning vertices in single graphs [111], our objective is notoriously more challenging because of the need to match substructures. This setting is very reminiscent of the issues for which DL solutions apply.

Our evaluation contemplates two metrics: silhouette score (SS) and adjusted rand index (ARI). The first measures how similar an event is to its own cluster (cohesion, i.e., intra-cluster distance) compared to the others (separation, i.e., nearest-cluster distance). The second computes the corrected-for-chance similarity between two event clusterings, counting pairs that are assigned in the same or different clusters in the predicted and true clusterings. Both lie in the range [−1,1], where high values indicate better results. In our study, we use the Euclidean distance. Rand index calculation is performed considering the best-predicted clustering upon ten consecutive runs of the K-means algorithm (scikit-learn implementation, with k=#event_types or k=#datasets). Experimental results are presented in Table 4.

#### 5.2.1. Results

At the outset, we can see that our solution performs the best on both metrics. For ease of understanding, next, we report percentage values. As for ARI, DDEGK averagely surpasses SciBERT, node2vec, and graph2vec by more than 10.7%, 14.3%, and 11.5%, respectively. As for SS, the gap settles on 3.4%, 23.3%, and 3.8%. This reinforces the findings inferred from the classification experiments; the scores reached by models on the clustering task are in line with that of classification. Significantly, clustering makes the impact deriving from a different anchor choice evident. The selection of random anchors per event type leads to a space with better geometric characteristics but less effective clustering than the entirely random counterpart.

#### 5.2.2. Influence of Embedding Dimensions

In the concrete, finding the optimal embedding dimension is not easy [16]. A longer embedding tends to preserve more information about the original event graph at the cost of more storage requirement and computation time, but it also risks maintaining noise. On the other hand, a lower dimension representation is more resource-efficient but risks losing critical information about the input graph with a significant performance drop. Researchers need to make a trade-off based on the requirements; GRL articles usually report that an embedding size between 128 and 256 is sufficient for most tasks. We study the effect of sub-sampling the dimensions of our embedding space on the quality of event graph classification and clustering.

**Table 4:** Between-graph clustering results according to the silhouette score (white row) and range index (gray row). The best results for each dataset are shown in bold.

Method	Event Type Clustering											Dataset Clustering
	ST09	GE11	EPI11	ID11	MLEE	GE13	CG13	PC13	GRO13	BIO_ALL	AVG	
SciBERT (AVG)	−0.00915	−0.00940	0.04082	0.01347	−0.01139	−0.00784	0.00087	−0.01251	−0.00343	**−0.01974**	−0.00183	−0.01123
	0.03638	0.06641	0.06644	0.09005	0.05810	0.04079	0.06644	0.04022	0.07669	0.04075	0.05823	0.05373
node2vec (AVG)	−0.48715	−0.48165	−0.58339	−0.58004	−0.46614	−0.57659	−0.51334	−0.49366	−0.54964	−0.58171	−0.53133	−0.27637
	−0.02987	−0.02539	−0.00234	−0.02405	−0.05510	−0.03748	−0.04139	−0.03689	−0.03148	−0.03830	−0.03223	0.00068
graph2vec	−0.25687	−0.20579	−0.14446	−0.27879	−0.35015	−0.28666	−0.39072	−0.36219	−0.41060	−0.45318	−0.31394	−0.16632
	0.03789	0.04679	0.09058	0.14508	0.03394	0.07483	0.02883	0.02123	0.01443	0.02412	0.05177	0.02761
DDEGK (*ours*)												
w/random anchors	0.21371	0.17281	0.27985	0.09267	0.08572	0.22813	−0.05122	−0.04587	0.05914	−0.32140	0.07108	**0.00686**
	0.33177	0.34138	**0.54129**	**0.44621**	**0.16441**	**0.51622**	**0.17242**	**0.13066**	**0.39070**	**0.14349**	**0.31786**	0.14349
w/random anchors per type	**0.23428**	**0.24598**	**0.28723**	**0.15141**	**0.10136**	**0.31270**	**0.08123**	**0.01809**	**0.08047**	−0.29036	**0.12224**	0.00007
	**0.35625**	**0.38622**	0.39765	0.29661	0.15441	0.43387	0.15185	0.09296	0.23732	0.08320	0.25903	**0.28101**

**Figure 8:**
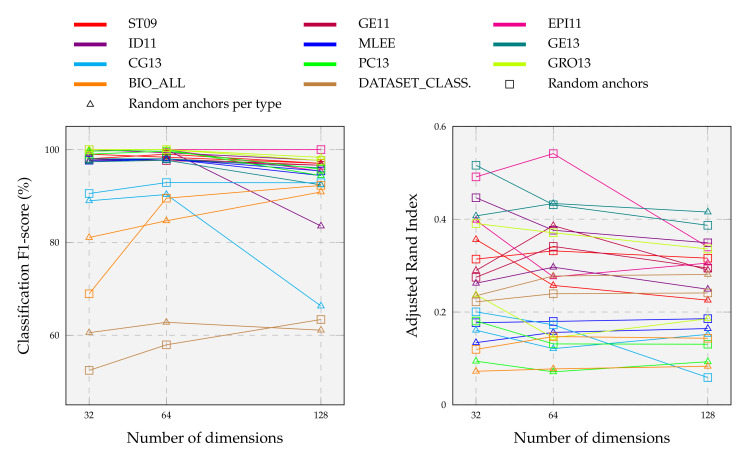
Effect of sub-sampling source graphs on event graph classification and clustering tasks above each biomedical dataset (colored line). We vary the number of source graphs between 32, 64, and 128, employing two different anchor strategies (marks).

By performing multiple tests on three bioinformatics datasets with an average of 570 instances, the authors of DDGK [19] achieved stable and competitive results using less than 20% of the graphs as anchors. To complement these findings, we explore computational complexity and scalability in much more detail. We construct different anchor graph sets from the original ones, adopting the anchor selection strategies listed in Section 5.1.2. For each configuration, we learn divergence scores against all target graphs and use the reduced embeddings as features to predict graph categories and clusters. Results are summarized in Figure 8.

We observe that classification scores on individual datasets remain substantially unchanged as dimensions vary with a random anchor choice. Only about a 2% drop in performance when the embedding dimension size drops from 128 to 32. On average, the highest F1-score for event type classification is obtained with an embedding size of 64, meaning that a high number of anchors is not required. So, very few dimensions are needed to obtain our final results detailed in Section 5.1 (far fewer than those expected by baselines). A higher number of dimensions is instead necessary for the more complex task of classifying heterogeneous event graphs. In this case, performance grows in step with embedding dimensionality, where more detailed representations capture several input nuances. Unlike a fully-random choice, anchors balanced with respect to the event type tend to perform worse with increasing dimensions, sometimes also experiencing substantial declines as in the case of CG13 and ID11. Relating the classification and clustering results facilitates understanding the overall graph embedding goodness and the correct hyperparameters setting. For example, DDEGK achieves high classification accuracy on PC13, but ARI is worse; while, on EPI11, despite the classification being perfect with all dimensions, the encoding space at 64 appears better at the clustering level.

### 5.3. Visualization

**Figure 9:**
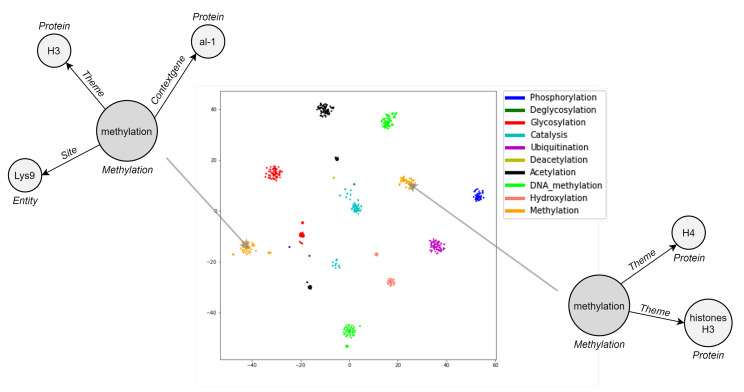
Embeddings of 1002 biomedical events from the EPI11 dataset, projected into a 2D space by t-SNE. Event graphs belonging to the same type are embedded closer to each other. In the case of structural or semantic differences, there can be multiple clusters for the same event type, as qualitatively shown for Methylation.

Embedding visualization is helpful to qualitatively realize how well DDEGK learns from the event graph structure and node/edge attributes. Since our representations capture events’ similarities by vicinity in the Euclidean space, the performance of event graph embeddings can be visually evaluated by the aggregation degree of labeled events (colorful dots). Ideally, vectors for event graphs in the same class should aggregate highly in a local region of the embedding space. Meanwhile, event vectors belonging to different classes should be as separable as possible to bolster downstream machine learning models. Hence, we conduct a visual intuitively inspection of how biomedical event graphs are grouped in the t-SNE 2D projection. For space reasons, we only show the plot of the two-dimensional manifold associated with EPI11, the best-encoded space in terms of clustering indices (Figure 9). DDEGK embeds event graphs that have similar types nearby in the embedding space, giving nicely distinguishable clusters. Notice that there may be different clusters for the same event type, oftentimes due to different structure or attribute sets. An event type within a schema can specify the cardinality of the expected participants, whose instances may sometimes be optional or multiple. It follows that events of the same type may have a very varied structure.

**Figure 10:**
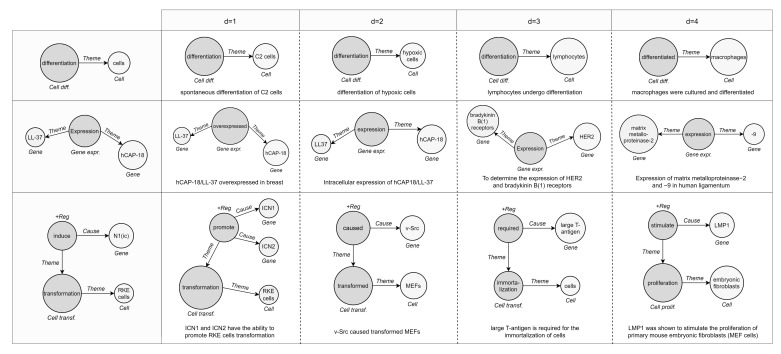
Query case studies on biomedical event graphs of different types and sizes. In each demo, the first column depicts the query and the others the similarity ranking of the retrieved graphs (and their mention). DDEGK correctly returns similar events in the structure or semantics of nodes/edges, also managing synonyms and acronyms thanks to SciBERT.

In Figure 10, we demonstrate three examples of event retrieval. The first shows a simple case where the query is unbounded, meaning that it contains an entity with a vague description “cells”, and the graphs returned detail that participant. In the second and third examples, the most similar events respect the structure and attributes of the reference (same types of participants and role played in the interaction), suggesting related or additional entities. Accordingly, it can be assumed that similar events refer to events with high-level semantic relationships rather than just identity relationships.

### 5.4. Cross-Graph Attention

**Figure 11:**
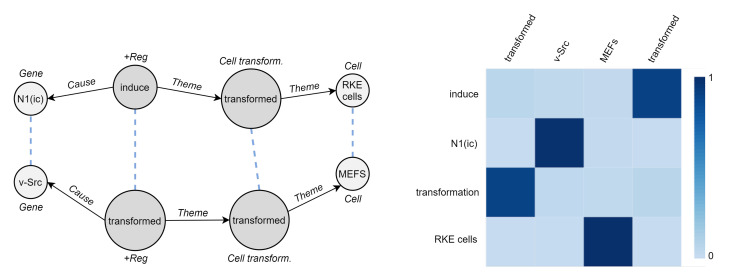
Qualitative example of DDEGK cross-graph attention, comparing two biomedical event graphs from CG13. Attention weights are visualized as a heatmap, while stronger node–node alignments are directly reported on the event graphs (dashed blue lines).

We seek to understand how two biomedical event graphs are related to each other by DDEGK. Since there is no gold-standard correspondence between the two sets of nodes, we cannot quantitatively evaluate the goodness of the cross-graph alignments. However, the qualitative example in Figure 11—which is not intended as a conclusive evaluation of the system—show how DDEGK typically pays the most attention to the correct entities or triggers while ignoring the irrelevant ones.

### 5.5. Semantic Textual Similarity

Language is highly ambiguous, with multiple ways to express the same concept unit, frequent high-level linguistic phenomena, and untold background knowledge. The superficial organization of a sentence is almost irrelevant for the identification of its real and deeper semantic content, determined instead by techtogrammatics [112]. Events allow us to remove noise and focus only on unambiguous relational knowledge involving relevant entities. To take a step in the research direction of comparing events and plain sentences within NLP applications, we also include an experiment on STS.

We consider three datasets on biomedical sentence similarity: BIOSSES [113], MedSTS [114], and CTR [115], for a total of 271 sentence pairs (499 unique sentences) and normalized scores in [0,1]. We process each sentence with seven pre-trained DeepEventMine models (GE11, EPI11, ID11, MLEE, GE13, CG13, PC13) [91], managing to extract 184, 8, 142, 331, 198, 392, and 236 events, respectively. In addition, 324 sentences have at least one event (264 with more than one), yielding a dataset of 127 sentence pairs (80:20 train-test ratio), event counterparts, and similarity scores. Subsequently, we convert events into event graphs and calculate entity embeddings as described in Section 4.1.1 and Section 4.2. By removing duplicated instances, we move from 1491 to 890 event graphs. Then, we apply DDEGK on the event graph population (128 random anchors, 4 node embedding dimensions, 3 encoding layers, 300 encoding and scoring epochs, $10^{-1}$ learning rate, [7,7,7] $\gamma^{v}$, $\tau_{v}$, $\tau_{e}$ preserving loss coefficients), with a mean pooling in case of multiple events per sentence. Finally, we calculate semantically meaningful sentence embeddings with SBERT [116] (all-mpnet-base-v2 from HuggingFace, https://huggingface.co/sentence-transformers/stsb-mpnet-base-v2, accessed on 11 December 2021).

We define a simple regression model composed of two fully connected linear layers (hidden size 1000) with a sigmoid in between, using the Adam optimizer, the L1 loss, 1000 epochs, and a learning rate equal to $10^{-4}$. We train it on three input embedding configurations: (i) sentences only (SBERT), (ii) events only (DDEGK), (iii) sentences + events (concatenated in this order). Remarkably, we register a mean squared error on the test set of 0.1379 for (i), 0.1216 for (ii), and 0.1598 for (iii). These results corroborate the effectiveness of events in grasping the essential knowledge mentioned in a text document. DDEGK event embeddings lead to better performance on downstream STS tasks than state-of-the-art approaches based on token sequences, using 6x lower dimensionality.

## 6. Discussion

Here, we briefly discuss findings and their implications, DDEGK limitations, and applicability to new scenarios.

DDEGK is an unsupervised and inductive method capable of mapping events into low-dimensional vectors, reflecting their structural and semantic similarities. It is designed to be highly interpretable, with a cross-graph isomorphic attention mechanism trained to preserve node and edge attributes. By merging DL architectures and NLP with symbolic structures and graph theory, it represents a powerful tool for automatically recognizing similarities between biomedical interactions mentioned by researchers in scientific publications, enabling their aggregation, quantification, and retrieval. It leverages deep graph kernels, but, as experimentally verified, it does not require computing the entire kernel matrix, providing greater scalability. Thus, learned embeddings make event-centered operations simpler and faster than comparable operations on graphs. We also argue that the representation of scientific knowledge in the form of events instead of textual documents can constitute a promising research direction for the performance improvement of different NLP tasks, especially when combined with memory-equipped neural networks [117,118,119] and deep metric learning [120].

On the other side, there are also some problems and limits. If it is true that non-individual representation learning leads to greater accuracy in estimating event similarity, it is equally true that kernel-based approaches are more time-consuming than other types of embedding. Furthermore, the choice of anchor graphs is crucial for the solution’s effectiveness and could be wisely carried out through an ad-hoc model. More specifically, we believe that a basis approximation could be obtained with a neural network [121], choosing as anchor events those whose weights are more orthogonal to each other and capture maximum variability; we leave this possibility as a future direction. Non-supervision based on comparison with some references makes it possible to calculate valid event embeddings even in the absence of paired datasets with annotated scores. However, it tends to bring out higher-level similarities than supervised approaches, which are instead conceivably capable of capturing differences at a greater level of detail. In addition, extending the attention mechanism beyond the node-to-node alignment could improve results and better recognize how two events are similar. Lastly, events may be accompanied by modifiers that reshape the interaction meaning, like negation (e.g., “not”) and speculation (e.g., “may”, “might”, “could”). Such properties have not been considered within this paper, but they can be seen as additional trigger attributes to boost performance.

DDEGK is highly flexible and can be easily applied to new datasets, tasks, and domains, possibly even on generic labeled graphs. For instance, one of the domains on which we pour more expectations is analyzing social posts shared by patients [122,123,124,125], e.g., detect conversational threads [126], aggregate and quantify the number of times an adverse drug reaction or symptom manifestation is reported under certain conditions. Four other applications that can benefit from event-enriched knowledge modeling are text classification [127], long document summarization [128], tutoring [129,130] and recommender [131,132] systems. Deploying our method on a new dataset essentially takes three steps: (i) select a pre-trained language model to encode node and edge text attributes; (ii) define a strategy for selecting a population subset with anchor role (which size in principle depends on graph characteristics and task complexity); and (iii) train a graph encoder for each anchor and use them to calculate target-anchor divergences from which derive whole-graph embeddings. As for (iii), the encoder architectural complexity and training duration should be commensurate with graph sizes. The intent is to avoid overfitting within the anchor graph encoder, which would otherwise reduce its usefulness in recognizing similar target graphs. In the datasets covered by this study, the best results were obtained with encoder layers and encoding/scoring epochs set to 3 and 300. The coefficients that regulate the impact of structure and semantics (i.e., discrete and continuous attributes of nodes and edges) should be adjusted according to their meaning for the specific application under consideration, possibly making them a training target. For example, in the case of biomedical events, the best setting was obtained by assigning the semantics a weight 21× greater than the structure and giving equal importance to node types, edge labels, and node textual attributes.

## 7. Conclusions

This paper presented Deep Divergence Event Graph Kernels, an unsupervised technique for learning whole-graph representations of biomedical events and their similarities. Our method compares the event graphs against a set of anchor ones without requiring feature engineering. Based on their structural and semantic divergence, also measured by SciBERT, it learns task-agnostic embeddings and a graph kernel above them. Using an isomorphic attention mechanism, we align nodes of two graphs without requiring a known correspondence between vertex ids, but only preserving the structure and consistency of node/edge attributes (both discrete and continuous). Our experimental analysis shows that, despite being trained with only graph edges, the learned representations encode numerous local and global information, resulting in a powerful embedding space. Moreover, when learned event embeddings are used as features in tasks like graph classification and clustering, we find them superior or competitive with those produced by other state-of-the-art solutions (even supervised). Furthermore, the excellent recognition of the semantic similarity between biomedical sentences highlights how incorporating dense and compact event-based features in NLP systems may be a promising research direction. Ultimately, in addition to being expressive, DDEGK models are incredibly informative. The control over anchor events and the presence of cross-graph attention weights allow a high level of insight into the alignment between two event graphs, making their similarity interpretable.

As future work, we will deepen (i) the automatic identification of anchor events orthogonal to each other, (ii) the passage from nodes to subgraphs as alignment granularity, (iii) the creation of multi-modal spaces combining events and text, (iv) the integration of events and language models, (v) deep neural networks with event-based memories, and (vi) the application to events not mentioned in the biomedical literature but expressed by patients and caregivers within social posts.

## Figures and Tables

**Table 2:** Hyperparameter values tested during our grid search for DDEGK graph representations.

Hyperparameter	Values
Node embedding	2, 4, 8, 16, 32
Encoder layers	1, 2, 3, 4
Learning rate	$10^{-4}$, $10^{-3}$, $10^{-2}$, $10^{-1}$, 1
Encoding epochs	100, 300, 600
Scoring epochs	100, 300, 600
$\gamma^{v}$, $\tau_{v}$, $\tau_{e}$ preserving	{7, 7, 7}, {8, 8, 4}
loss coefficients	{10, 6, 4}, {15, 0, 5}

**Table 3:** Average accuracy (support-weighted F1-score) in ten-fold cross validation on event type and dataset classification tasks. Methods are grouped by their approach and level of supervision during graph representation learning. The highest F1-score for each dataset is shown in bold.

Method		Unsupervised	Event Type Classification											Dataset Classification
			ST09	GE11	EPI11	ID11	MLEE	GE13	CG13	PC13	GRO13	BIO_ALL	AVG	
SciBERT	AVG	✓	72.16	73.24	89.55	85.45	72.18	82.53	69.50	72.59	78.08	58.72	75.40	53.11
	SUM	✓	56.32	58.91	94.34	76.97	58.35	81.02	52.68	53.51	65.35	42.63	64.01	47.68
	MAX	✓	64.54	53.95	90.47	75.95	56.16	74.35	63.91	60.39	72.16	51.11	66.30	49.22
BioBERT	AVG	✓	71.43	70.11	90.60	85.40	71.91	79.81	66.77	70.40	76.72	54.59	73.77	52.61
	SUM	✓	61.49	58.73	93.32	73.22	56.03	76.94	56.36	56.53	59.60	43.64	63.59	50.17
	MAX	✓	56.38	53.95	90.47	75.95	56.16	74.35	57.26	56.86	76.72	45.72	64.38	51.82
node2vec	AVG	✓	19.65	21.03	23.81	14.18	18.58	19.24	7.51	12.60	9.91	11.38	15.79	14.05
	SUM	✓	25.90	23.43	23.71	22.05	15.85	19.37	13.62	15.19	8.89	8.68	17.67	14.31
	MAX	✓	28.13	22.06	24.31	29.26	16.45	22.80	13.00	15.42	14.91	11.21	19.76	15.20
node2vec-PCA		✓	26.71	24.17	32.04	31.06	22.53	34.43	17.90	24.51	23.16	16.54	25.31	18.26
graph2vec		✓	54.12	58.60	57.78	62.25	41.34	63.47	44.59	40.51	33.81	40.48	49.70	43.06
DGCNN			89.19	89.04	90.40	88.70	93.19	86.65	95.73	93.35	94.23	89.55	91.00	**89.55**
DDEGK (*ours*)														
w/random anchors		✓	99.00	**97.99**	**100**	**100**	98.01	**98.02**	**92.89**	**99.67**	**100**	**92.28**	**97.86**	63.39
w/random anchors per type		✓	**99.01**	**97.99**	**100**	**100**	**98.02**	97.70	90.32	**99.67**	**100**	90.87	97.36	62.79

## Data Availability

For replication purposes, code and datasets are publicly available at https://github.com/disi-unibo-nlu/ddegk, accessed on 11 December 2021.
